# Users’ continuance intention towards an AI painting application: An extended expectation confirmation model

**DOI:** 10.1371/journal.pone.0301821

**Published:** 2024-05-15

**Authors:** Xiaofan Yu, Yi Yang, Shuang Li

**Affiliations:** 1 Postdoctoral Research Station, Central Academy of Fine Arts, Beijing, China; 2 School of Art, Soochow University, Suzhou, China; Universidad Central de Chile, CHILE

## Abstract

With the rapid advancement of technology, Artificial Intelligence (AI) painting has emerged as a leading intelligence service. This study aims to empirically investigate users’ continuance intention toward AI painting applications by utilizing and expanding the Expectation Confirmation Model (ECM), Technology Acceptance Model (TAM), Unified Theory of Acceptance and Use of Technology (UTAUT), and the Flow Theory. A comprehensive research model is proposed. A total of 443 questionnaires were distributed to users with AI painting experiences for data collection. The hypotheses were tested through structural equation modeling. The primary conclusions drawn from this research include: 1) Confirmation plays a crucial role, significantly and positively predicting satisfaction and social impact. 2) Personal innovativeness has a significant effect on confirmation. 3) Satisfaction, flow experience, and social influence directly and positively predict intention, with social influence showing the most significant impact, while perceived usefulness, perceived enjoyment, and performance expectancy show no significant impact on intention. 4) Habit plays a negative moderating role in the association between social influence and continued intention to use. These findings offer valuable insights and inspiration for users seeking to understand the appropriate utilization of AI painting and provide actionable directions for the development of AI painting.

## 1 Introduction

Today, artificial intelligence (AI) has permeated every aspect of our lives, including communication, innovation, social activities, and even painting. AI painting gains widespread popularity among internet users worldwide. The integration of AI technology into the realm of art can provide users with new opportunities for artistic creation [1]. The advantages of using AI painting tools are undeniable, offering users convenience, openness, sharing capabilities, efficiency, and time-saving benefits. In 2022, the popularity of AI painting soard. Platforms like TikTok, Little Red Book, AI Art, and Assisted Creation Platform have made neural style transfer, a technique that combines AI technology and art, common practice [2]. The topic of "AI painting" on TikTok alone garnered nearly 1.3 billion views, with over 30 million people actively engaging with it. As the number of AI painting users continues to grow, its potential for promoting artistic activity becomes increasingly promising.

Existing research on AI painting uses different theories, including the technology acceptance model [3], and the unified theory of acceptance and use of technology [4], to explain users’ adoption of AI painting. However, the findings are still fragmented. Previous studies on AI painting primarily focused on the generation systems [5, 6] and art education [7]. Although general-purpose AI painting tools found more applications in fields like art education and design, it is essential to take the continuing intention to use into consideration when promoting this new technology. The majority of prior studies on AI painting devices aimed to investigate users’ initial utilization of the devices, while only a few studies have examined their behavior and intention to continue using them.

Previous studies provided empirical evidence that demonstrates the moderating effects of users’ habits on artificial intelligence (AI) technology products and services, leading to increased intentions to repurchase [8], this effect also extends to AI Chatbots [9]. However, these findings are not investigated in the context of AI painting. Additionally, social media habits reflect automatic expressions of past behavior, aligning with social psychology and occurring within a stable environment, as noted in previous studies [10]. Habit plays a crucial role in predicting future purchasing and usage behaviors. When individuals use AI painting in a stable environment, they develop refined usage patterns through ongoing interactions, fostering an emotional connection and affirmation towards AI painting. Usage habits result from repeated patterns and goal-oriented behaviors over an extended period. Habit influences usage behavior in an interactive relationship [11]. Therefore, this study recognizes habits as a potential moderating variable.

Thus, this study aims to examine users’ post-continuance behavior concerning AI painting. To address this issue, the present study incorporates the Expectation Confirmation Model (ECM), Technology Acceptance Model (TAM), Unified Theory of Acceptance and Use of Technology (UTAUT), and the Flow Theory into an integrated user adoption model of AI painting. Previously, the Expectation Confirmation Model has been utilized to explore users’ perspectives on various technologies such as sharing economy platforms [12], mobile instant messaging, and Internet protocol television [13]. However, there are few attempts to apply this theory to understand users’ perspectives on AI painting. By applying the ECM to the integrated research model of AI painting, we can gain a more comprehensive understanding of users’ behaviors and usage patterns in the context of AI painting. Furthermore, the findings of this study can demonstrate the potential and feasibility of combining multiple theories in proposing an integrated research model for users’ continuance intention to use behavior.

The remainder of the paper was structured as follows: Section II provides a brief overview of AI painting and reviews related literature and the development of hypotheses. Section III describes the methodology employed to examine the proposed model. Section IV describes the results of the proposed model. Finally, Section V presents and discusses the research results and their implications.

## 2 Literature review and theoretical models

### 2.1 AI painting

AI painting has gained significant popularity in China, with many users embracing this tool for various purposes [14]. It serves as a helpful assistant in people’s daily lives, enabling them to enhance their painting experiences with just a click on their phones. The convenience of generating AI paintings and the ability to save and share the created images permanently have contributed to its widespread use [15].

The increasing utilization of AI painting has attracted academic attention, leading to numerous studies exploring its significance and users’ attitudes towards it. For instance, Sun et al. (2022) compared human and AI paintings using a deep neural network (DNN) [16]. Yu et al. (2022) analyzed paintings generated by word-to-image systems and investigated users’ creative actions and attitudes toward AI through experiments [2]. Gu. et al. (2022) examined how AI art has influenced the valuation, purchase, and collection intentions of Chinese and Western paintings [17]. Furthermore, researchers have focused on exploring the adoption intentions of AI painting software and understanding the factors that drive user behavior. Previous studies applied models such as ETAM (Expectation Confirmation Model) and AIBPS (Artificial Intelligence-based Behavior Prediction System) to examine the application of AI painting and identify positive and negative factors associated with perceived usefulness [18]. Another study by Du et al. in 2023 examined the factors influencing the attitudes and behavioral intentions of designers from different fields in using AI painting tools [4].

### 2.2 Expectation confirmation model

The Expectation Confirmation Model (ECM) is a widely used theory in the field of marketing that focuses on evaluating customer satisfaction and understanding post-purchase behavior [19]. It also applies to assess users’ continued intention to use information systems [20]. The theory aims to provide a comprehensive analysis of the consumer consumption process [21]. In the context of AI technology, several researchers have utilized the ECM framework to understand user behavior and satisfaction [4, 20].

In the expectation confirmation model, the confirmation of expectations plays a crucial role in shaping user satisfaction [22]. By comparing their perceived satisfaction with initial expectations, users can assess the alignment or divergence between the two [23, 24]. User satisfaction is determined by the discrepancy between pre-purchase expectations and confirmed expectations through post-purchase evaluation [25]. However, when it comes to the continued use of AI painting, user behavior becomes an important factor influencing decision-making. Therefore, theoretical extensions may be necessary to explain the factors affecting the ongoing use of AI painting tools.

#### 2.2.1 Satisfaction

Satisfaction (SAT) refers to the psychological structure that corresponds to the user’s response to the level of satisfaction, indicating whether the satisfaction is positive or negative [21]. Satisfaction has long been used to study the continuance intention to use different types of online products or services [26–28]. For instance, Prathap (2020) found that users’ perceived satisfaction in using mobile payments plays a determining role in their intention to continue using them. In the context of AI painting [29], if users’ experience exceeds their expectations, they are likely to feel satisfied, leading to a positive inclination for continued usage of AI painting. Consequently, the following assumptions were made:

**H1:** Satisfaction is positively related to user continuance intention with the AI painting.

#### 2.2.2 Confirmation

Confirmation (CON) refers to the alignment between an individual’s perception of the outcome and their established expectations [30–32]. Confirmation was recognized as an influential factor in various aspects, such as perceived usefulness, perceived ease of use, perceived enjoyment, effort expectancy, and social influence, in previous studies [25, 26]. Cheng (2021) studied the concept of confirmed and satisfaction in robo-advisor services [33]. The results show that a higher level of positive certainty means higher satisfaction. However, interestingly, Park (2020) found that users’ confirmation had a non-significant impact on satisfaction with smart wearable devices [34]. Furthermore, the positive correlation between confirmation, perceived usefulness, and perceived ease of use is observed in the context of smartphones being used as smart pedagogical tools [35]. Therefore, it is expected that confirmation will influence the post-use experience of AI painting, including users’ satisfaction, perceived usefulness, perceived ease of use, perceived enjoyment, effort expectancy, and social influence. Based on this, the following six hypotheses are proposed:

**H2:** Confirmation is positively related to satisfaction with AI painting.
**H3:** Confirmation is positively related to the perceived usefulness of AI painting.
**H4:** Confirmation is positively related to the perceived ease of use of AI painting.
**H5:** Confirmation is positively related to perceived enjoyment of AI painting.
**H6:** Confirmation is positively related to the effort expectancy of AI painting.
**H7:** Confirmation is positively related to the social influence of AI painting.

#### 2.2.3 Personal innovativeness

Personal innovativeness (PI) is defined as the consistent and determined use of a specific new information technology innovation by an individual [36]. Within the realm of information technology, the personal innovativeness exhibited by a user plays a significant role in influencing innovative behavior. Some researchers have identified personal innovativeness as a crucial antecedent [37, 38], and others have recognized it as a significant moderating factor [39, 40].

Numerous studies established a connection between personal innovativeness and user satisfaction, showing that an individual’s level of innovativeness greatly impacts his or her willingness to adopt new systems or technologies. Huang et al. (2007) expanded the expectation confirmation model by incorporating personal innovativeness as one of the individual factors [41]. The study by Lee et al. (2021) also found that personal innovativeness is positively correlated with use confirmation in the context of artificial intelligence-based voice assistant systems [42]. Consequently, for the use of AI painting applications, it is expected that individuals with high levels of personal innovativeness will actively interact with various features, explore newly introduced applications and services, and thereby strengthen their confirmation to a greater extent compared to those with low personal innovativeness. Therefore, the present study puts forward the following hypothesis:

**H8:** Personal innovativeness is positively related to the confirmation of AI painting.
**H9:** Personal innovativeness is positively related to the satisfaction of AI painting.

### 2.3 Technology acceptance theory

The Technology Acceptance Model (TAM), initially proposed by Davis in 1986 [43], has become a widely used framework for understanding and predicting user behavior in the adoption of information technology and systems [44, 45]. The theory aims to analyze the influence of external factors on personal beliefs, attitudes, and intentions [46]. TAM has been applied in various fields such as technology, marketing, AI robotics, and psychology to examine online consumer behavior and technology adoption [3]. The key concept in TAM is that perceived ease of use and perceived usefulness significantly impact users’ attitudes and intentions to continue using a particular technology, thereby influencing their actual use behavior.

The Unified Theory of Acceptance and Use of Technology (UTAUT) is an extension of TAM that integrates relevant models from the 1990s [47]. It consists of four components: effort expectancy, performance expectancy, social factors, and facilitating conditions, along with four moderating factors: age, gender, education, and voluntariness of use. The combination of these constructs and moderating factors directly influences the assessment of behavioral intention. This study adopts the UTAUT model, focusing specifically on the factors of perceived enjoyment, effort expectancy, and social influence.

Both the TAM and UTAUT models are widely used to assess technology acceptance [48]. With the emergence of AI in recent years, new products, services, applications, and systems have been introduced to the market, providing customers with access to a wide range of AI-powered technologies. In the context of AI painting, this study focused on users’ intentions to continue using AI painting tools, which represent their decision to continue using the technology after initial adoption. Understanding users’ continued usage behavior is an essential aspect of technology acceptance and adoption [20].

#### 2.3.1 Perceived usefulness

Perceived usefulness (PU) refers to users’ subjective assessment of the utility of AI painting experiences [49]. Numerous studies have identified perceived usefulness as a key factor influencing users’ satisfaction with technology and their intentions to persist in its use [50, 51]. Oyman et al. (2022) have suggested that users’ perceived usefulness plays a significant role in the willingness to use augmented reality technologies [52]. This study focuses on AI painting continuance intentions to use, defined as users’ decision to continue using AI painting technology after accepting it. We therefore propose the following assumptions:

**H10:** Perceived usefulness is positively related to user satisfaction with AI painting.
**H11:** Perceived usefulness is positively related to users’ intention to continue using AI painting.

#### 2.3.2 Perceived ease of use

Perceived ease of use (PEOU) refers to an individual’s subjective assessment of the simplicity and effortlessness involved in utilizing a particular system or technology, taking into account aspects like the ease of learning and the minimal physical and mental exertion needed [39]. A multitude of research have validated that perceived ease of use affects satisfaction with using new technologies and is a determinant of users’ overall satisfaction. Previous studies have consistently shown a positive correlation between perceived ease of use and consumer satisfaction regarding the use of information systems and services [41]. The findings of Ngubelanga and Duffett (2021) suggested that perceived ease of use has a reinforcing effect on consumers’ satisfaction with mobile commerce applications [50]. Thus, we hypothesize that:

**H12:** Perceived ease of use is positively related to user satisfaction with AI painting.

#### 2.3.3 Perceived enjoyment

Perceived enjoyment (PE) refers to the pleasure or enjoyment experienced by individuals when using AI painting [53]. In a study by Davis et al. (1992), enjoyment variables were added to the TAM model to explain the influence of intrinsic motivation on the adoption of new technologies [54]. Previous research has affirmed the pivotal role of perceived enjoyment in shaping behavioral intentions toward the acceptance and utilization of diverse information systems and technologies [52, 55]. The concept of perceived enjoyment has been extensively applied in the realm of users’ adoption of AI, notably within network environments [42]. In their research, Yang and Lee (2019) establish that perceived enjoyment is a crucial determinant in the users’ willingness to use virtual personal assistant devices [56]. Drawing from the available literature, the following hypothesis was formulated:

**H13:** Perceived enjoyment is positively related to users’ intention to continue using AI painting.

#### 2.3.4 Effort expectancy

Effort expectancy (EE) is defined as the perception of the degree of ease associated with using a system or technology [47]. Numerous empirical studies have proven that effort expectancy is found to influence behavioral intention [26, 57]. Adjei et al. (2021) found a positive relationship between effort expectancy and the intention to use mobile phone-based Interactive Voice Response in a rural area of Ghana [58]. In contrast, Qin and Yu (2023) identified that effort expectancy negatively influenced the intention to use Tencent Meeting for online courses [59]. Hence, additional research is needed to these inconsistencies. As a result, the following assumption was proposed:

**H14:** Effort expectancy is positively related to users’ intention to continue using AI painting.

#### 2.3.5 Social influence

Social influence (SI) pertains to the degree to which a user believes that others, particularly their acquaintances and friends, significantly impact their decision to continue using new technologies [47]. Studies have shown that social influence has a significant effect on people’s readiness to adopt new technologies [60]. Additionally, social influence is a key factor in behavioral intention across various artificial intelligence studies involving AI tools [61], AI Integrated CRM Systems [62], and AI-assisted learning environments [63]. Zeebaree et al. (2022) observed a positive correlation between social influence and the intention to adopt E-Government services in northern Iraq [64]. Conversely, Kašparová (2023) discovered that social influence hurt the behavioral intention towards business intelligence tools [65]. Therefore, further research is necessary to address these discrepancies. Consequently, the following assumptions were made:

**H15:** Social influence positively related to user continuance intention of AI painting.

### 2.4 The flow theory

Flow theory suggests that a positive subjective experience is a key driver for engaging in an activity [66]. When individuals "feel good" about an activity, it has an intrinsic motivational effect, leading to increased involvement. In a state of flow, individuals are motivated by internal factors, experience a sense of control, and maintain focused concentration [67].

Flow experience (FLO) refers to the mental state of complete immersion and engagement in an activity [68]. People in a state of flow are fully absorbed in an activity, focused on a specific goal, and disregard the presence of other factors, resulting in feelings of happiness and fulfillment [69]. Flow experience is considered the most optimal state of experience for individuals and is used as a reliable measure of satisfaction in various domains, including travel and tourism, where it serves as a significant indicator of destination loyalty [70].

In the context of AI painting, it is important to consider how to maintain an optimal level of engagement to keep users in a state of flow. Based on this understanding, the following assumption was made:

**H16:** Flow experience is positively related to users’ intention to continue using AI painting.

### 2.5 Habits

Habits (HAB) are memory-based tendencies that automatically respond to cues and lead to repeated past behaviors [71]. Habits are part of the array of non-conscious processes that can influence behavior [72]. These tendencies develop through the associations formed between cues and responses in memory, which are solidified through repeated actions in consistent contexts [73]. Habits play a foundational role in shaping behavior, impacting decision-making processes, and reducing the reliance on conscious thought [74].

This study delves into how habits moderate the link between a social influence and the intention to continue, drawing on previous research [8, 75], that has highlighted the direct connection between habits and the intention to continue. Despite the crucial role of habits as moderators, they have been relatively overlooked in the artificial intelligence literature. Prior studies have shown that habits moderating effect influences user satisfaction, Perceived Usefulness, flow experience, and the intention to continue use [76]. Some research also indicates that habits have a slight negative moderating impact on the relationship between customer satisfaction and the intention to continue [77]. However, there is a scarcity of research that integrates habits with the Extended Expectation Confirmation Model (ECM) and TAM in the context of AI painting, implying that habits could potentially regulate the relationships among these constructs. Building on this insight, the following assumption is put forward:

**H17:** Habits play a moderating role in the relationship between users’ social influence and their intention to continue using AI painting.

### 2.6 Research model

**Fig 1:**
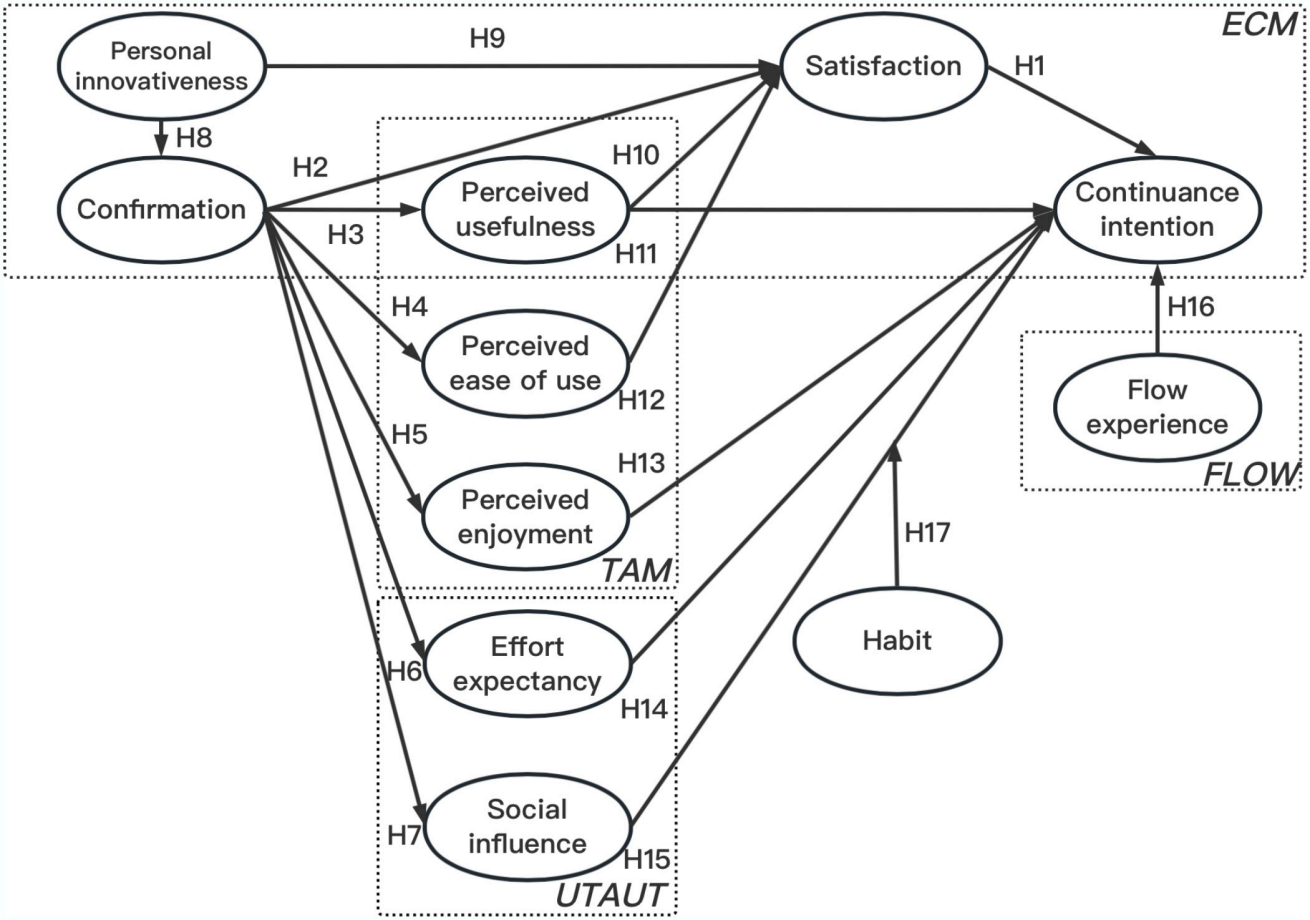
The hypothesized model.

Building on prior research, this study expanded the existing ECM (Extended Conceptual Model) by integrating multiple user-oriented theories such as TAM (Technology Acceptance Model), UTAUT (Unified Theory of Acceptance and Use of Technology), and the Flow model (refer to Fig 1). Moreover, the authors have introduced habits as a moderating factor within the model. Through the integration of these theories and the exploration of habits’ moderating influence, the study seeks to develop a thorough insight into users’ continuance intentions to use AI-assisted painting.

## 3 Methodology

### 3.1 Questionnaire design and measurements

The questionnaire design process proposed by Park (1993) serves as the foundation for this study [78]. The data collection started on May 18, 2023, and ended on September 29, 2023. We obtained written consent from participants in questionnaires, and we also got ethics approval from the ethics committee of the first author’s local institution (Effective Approval Date: 5 May 2023). To ensure the accuracy and comprehensiveness of the questionnaire, we followed the three-indicator rule, which required each structure to have at least three items [79]. The 34 questions representing the 11 structures were extracted primarily from professional journals. To accurately convey the content from the initial query posed in English, a dual translation approach (English to Chinese) was employed. The translation process was conducted by two professional translators, and the questionnaire was further revised by three relevant professors.

The questionnaire was divided into two sections. The first section captures participants’ background information, such as age, gender, and profession. The second section comprises a series of questions related to the study’s subject. Before answering the questionnaire, all participants had experience using AI for painting. Previous studies have recommended using a seven-point Likert scale as an interval scale, with scores ranging from 1 (strongly disagree) to 7 (strongly agree). This scale was employed to effectively assess all potential variables. After revising the questionnaire based on feedback, a pilot test was conducted with 30 participants, resulting in the elimination of four items and retaining the valid questions. The questionnaire’s structure, including the remaining items, is displayed in Table 1.

**Table 1:** Questionnaire items.

Construct	Item	Reference
Perceived ease of use	PEOU1: I found that TikTok AI painting technology is easy to master. PEOU2: For me, the operation interface of TikTok AI painting is very simple. PEOU3: For me, the use of TikTok AI painting does not require brainpower.	[49]
Perceived usefulness	PU1: Painting with TikTok AI will make my creation easier. PU2: Through to use of TikTok AI painting, my learning and knowledge in painting have been strengthened. PU3: TikTok AI painting can save me time in painting creation and improve efficiency.	[49]
Perceived enjoyment	PE1: I think the process of painting with TikTok AI is exciting. PE2: I like to use TikTok AI painting to create. PE3: I think the process of painting with TikTok AI is very pleasant.	[53]
Social influence	SI1: People who are important to me think I should use TikTok AI to paint. SI2: People familiar with me think I should use TikTok AI to paint. SI3: Most people around me have used TikTok AI painting.	[47]
Effort expectancy	PEX1: I found that using TikTok AI painting can enhance my creative inspiration. PEX2: TikTok use of AI painting technology will enable me to receive services more effectively. PEX3: I found that using TikTok AI painting can enhance my creative expression.	[47]
Satisfaction to use	SAT1: I am satisfied with the experience of TikTok AI painting. SAT2: I am satisfied with the function of TikTok AI painting. SAT3: I am satisfied with the overall use of TikTok AI painting.	[80]
Continuance intention to use	CITU1: I will often use TikTok AI for painting in the future. CITU2: I will regularly use TikTok AI for painting in the future. CITU3: I would strongly recommend others to use TikTok AI for painting.	[20]
Habit	HAB1: Using TikTok AI for painting has become a habit for me. HAB2: I am addicted to using TikTok AI for painting. HAB3: I must use TikTok AI to paint. HAB4: It’s natural for me to use TikTok AI for painting.	[81]
Flow experience	FLO1: Using TikTok AI to paint can make me lose focus on what’s happening around me. FLO2: When using TikTok AI for painting, I feel like I lose my sense of time and feel like time flies by quickly. FLO3: When I create TikTok AI paintings, I will be very focused.	[66]
Confirmation	CON1: I used TikTok AI to paint better than I expected. CON2: TikTok AI painting is more interesting than I expected. CON3: TikTok AI painting met my expectations.	[20]
Personal innovativeness	PI1: If I hear about a new painting creation technique, I will try every way to try it out. PI 2: Generally speaking, I prefer to actively try new painting techniques. PI 3: Among my peers, I am usually the first to try new painting techniques. PI 4: I enjoy trying new painting techniques.	[20]

### 3.2 Participants

The study was primarily conducted in Chinese cities, where there is a higher prevalence of AI painting within the TikTok app compared to other regions. Participation in the study required participants to have prior experience using AI painting. To ensure participants’ understanding of the subject matter, an overview of the definition of AI painting was provided before they answered the questionnaire. All participants used AI drawings to ensure their familiarity with the topic.

Before data collection, the researchers provided participants with a summary of the study, instructions for information gathering, and obtained informed consent. It is important to ensure that participants were fully informed about the purpose and procedures of the study, as well as their rights and privacy protection.

### 3.3 Collection and analysis

The online questionnaire was conducted in China using popular social media platforms such as Tencent QQ, WeChat, and Weibo. This approach was chosen to target individuals who frequently engage with the internet and technology, as they are more likely to exhibit an interest in technological items like AI painting. The web-based questionnaire was available for 30 days and aimed to assess the attitudes of 610 individuals towards AI painting.

After removing incomplete and unsuccessful responses from the attention tests, a total of 443 valid questionnaires remained for analysis. The demographic information of the research participants is presented in Table 2, providing an overview of the characteristics of the sample.

**Table 2:** Participants’ information.

Construct	Item	Frequency	Percent
**Gender**	male	180	40.6
	female	263	59.4
	total	443	100
**Age**	under 18	15	3.4
	18–30	349	78.8
	31–40	52	11.7
	41–55	25	5.6
	above 55	2	0.5
	total	443	100
**Career**	student	286	64.6
	office worker	79	17.8
	freelancer	30	6.8
	public official	8	1.8
	professional	24	5.4
	others	16	3.6
	total	443	100
**Education**	high school and below	26	5.9
	bachelor	233	52.6
	master	149	33.6
	doctor	35	7.9
	total	443	100
**Income**	under 2500	223	50.3
	2500–5000	88	19.9
	5001–10000	90	20.3
	above 10000	42	9.5
	total	443	100

To examine the interrelationships among variables, particularly in intricate models, Structural Equation Modeling (SEM) was employed. SEM requires a recommended ratio of 1:10 between the number of observations and the number of indicators. However, Bentler (1996) proposes an alternative guideline where the sample size should be five times larger than the number of free parameters [82]. Given the information provided, the sample size of 443 valid responses satisfies the prescribed threshold, as it falls within the recommended range of 170 to 340.

Before conducting the data analysis, the demographic information was summarized, and the statistical validity was verified using SPSS 26.0 software [83]. Additionally, to test the proposed hypotheses and examine the correlations between each variable, a path analysis was performed using AMOS 24.0 software. The researchers utilized the PROCESS plug-in to explore the impact of the moderator on the conditional effect, providing further insights into the relationships between variables.

## 4 Results

### 4.1 Preliminary analysis

Table 3 presents the initial analytical data within the study model. The table indicates that there are 443 valid samples for each item, with no missing data observed. Additionally, the standard deviation and mean values for all items fall within the normal range, implying that there are no data entry errors or anomalies.

**Table 3:** Descriptive analysis of items and constructs.

Item	Mean	Std. Deviation	Skewness	Kurtosis	Mean
PEOU1	3.73	0.958	-0.553	-0.139	3.731
PEOU2	3.75	0.876	-0.484	0.01	
PEOU3	3.72	0.989	-0.534	-0.149	
PU1	3.58	0.951	-0.573	0.055	3.475
PU2	3.25	1.07	-0.191	-0.598	
PU3	3.6	0.954	-0.525	-0.009	
PE1	3.42	0.946	-0.335	-0.156	3.4
PE2	3.23	0.994	-0.433	-0.312	
PE3	3.56	0.931	-0.575	0.292	
PEX1	3.42	0.968	-0.499	-0.142	3.514
PEX2	3.53	0.918	-0.529	0.031	
PEX3	3.58	0.944	-0.627	0.278	
SI1	2.99	0.974	-0.066	-0.423	3.083
SI2	3	0.993	-0.028	-0.466	
SI3	3.26	1.006	-0.391	-0.421	
HAB1	2.58	0.998	0.367	-0.574	2.489
HAB2	2.33	1.057	0.77	0.111	
HAB3	2.41	1.069	0.486	-0.549	
HAB4	2.64	1.117	0.239	-0.77	
FL1	2.93	1.072	-0.166	-0.83	2.759
FL2	2.59	1.078	0.362	-0.838	
PI1	3.62	0.893	-0.698	0.439	3.513
PI2	3.69	0.83	-0.537	0.402	
PI3	3.08	1.012	0.065	-0.793	
PI4	3.67	0.893	-0.536	0.165	
CON1	3.29	0.962	-0.427	-0.266	3.29
CON2	3.41	0.981	-0.441	-0.06	
CON3	3.17	0.99	-0.326	-0.452	
SAT1	3.45	0.914	-0.627	0.375	3.445
SAT2	3.42	0.942	-0.449	0.132	
SAT3	3.46	0.939	-0.648	0.421	
ITU1	3.04	1.046	-0.037	-0.507	3.067
ITU2	3.03	1.011	-0.024	-0.527	
ITU3	3.13	1.026	-0.244	-0.426	

To assess the normal distribution of the variables, it is recommended that the magnitudes of skewness and kurtosis remain below 1.5 [84]. The absolute values of skewness and kurtosis in the data have been found to meet this conventional criterion, suggesting a normal distribution of the constructs. This finding provides a solid foundation for future research endeavors, as it ensures the appropriateness of statistical analyses and interpretations.

### 4.2 Analysis of exploratory factors

To assess the validity and reliability of the measurement scales used in the study, certain criteria are commonly employed. The standard factor loading for each item should ideally be higher than 0.6, and at least above 0.5 to be considered acceptable [85]. In Table 4 the factor loading results range from 0.693 to 0.929, which are all above the 0.5 threshold. This indicates that the factor structure utilized in this study is suitable for examining the research objectives.

**Table 4:** Units for magnetic properties.

Item	Corrected Item-To-Total Correlation	Cronbach’s Alpha
PEOU1	0.820	0.772
PEOU2	0.841	
PEOU3	0.828	
PU1	0.870	0.801
PU2	0.855	
PU3	0.818	
PE1	0.855	0.849
PE2	0.889	
PE3	0.887	
PEX1	0.824	0.821
PEX2	0.874	
PEX3	0.878	
SI1	0.885	0.773
SI2	0.906	
SI3	0.693	
HAB1	0.905	0.924
HAB2	0.898	
HAB3	0.898	
HAB4	0.913	
FL1	0.854	0.630
FL2	0.854	
PI 1	0.825	0.816
PI 2	0.856	
PI 3	0.704	
PI 4	0.842	
CON1	0.922	0.899
CON2	0.895	
CON3	0.920	
SAT1	0.926	0.917
SAT2	0.923	
SAT3	0.929	
ITU1	0.921	0.894
ITU2	0.909	
ITU3	0.896	

For validity analysis, reliability is typically indicated by Cronbach’s alpha coefficient values greater than 0.7 [86]. However, it is also considered acceptable to consider a Cronbach’s alpha coefficient value of 0.6 as a "criterion-in-use" [87]. In Table 4, the Cronbach’s alpha values presented for each construct demonstrate internal consistency above 0.6, indicating acceptable reliability.

### 4.3 Validity test

To assess the convergent and discriminant validity of the measurement scales used in the study, Confirmatory Factor Analysis (CFA) provides useful indices. Comprehensive Reliability (CR) and Average Variance Extraction (AVE) should typically exceed 0.7 and 0.5, respectively [64]. Furthermore, to evaluate discriminant validity, the square root of the AVE for each construct should be greater than the correlations between constructs [88].

Based on the previous study, a total of 443 samples were analyzed, and the mean and standard deviation values for each construct are presented in Table 4. The values of CR and AVE for all constructs are higher than the respective thresholds of 0.7 and 0.5, indicating satisfactory convergent validity.

In Table 5, the diagonal entries represent the square root of the AVE, which is displayed in bold. These values are found to be higher than the correlations between the constructs, indicating discriminant validity. This outcome provides evidence that the measurement scales used in the research have achieved both convergent and discriminant reliability.

**Table 5:** Results of internal and convergent reliability.

Construct	Composite Reliability	Convergence Validity	Correlation And Discriminant Validity										
	CR	AVE	PEOU	PU	PE	PEX	SI	HAB	FL	SINN	CON	SAT	ITU
PEOU	0.869	0.688	**0.829**										
PU	0.885	0.719	0.314	**0.848**									
PE	0.909	0.769	0.317	0.696	**0.877**								
PEX	0.894	0.738	0.305	0.669	0.689	**0.859**							
SI	0.871	0.695	0.186	0.511	0.55	0.574	**0.834**						
HAB	0.947	0.816	0.038	0.451	0.506	0.443	0.635	**0.903**					
FL	0.843	0.729	0.04	0.378	0.401	0.425	0.485	0.637	**0.854**				
PI	0.883	0.654	0.239	0.334	0.428	0.506	0.412	0.367	0.346	**0.809**			
CON	0.937	0.833	0.296	0.595	0.613	0.579	0.51	0.56	0.502	0.48	**0.913**		
SAT	0.948	0.857	0.297	0.582	0.58	0.577	0.496	0.496	0.433	0.454	0.786	**0.926**	
ITU	0.934	0.826	0.127	0.581	0.582	0.507	0.588	0.66	0.516	0.426	0.636	0.615	**0.909**

The bold diagonal is the square root value of average variance extracted (AVE), and the lower triangle is the Pearson correlation of structures.

### 4.4 Fit indices

Assessing the goodness of fit of a model to the collected data is a common research practice. Various fit indices, including the Chi-square (X2) statistic, degrees of freedom (DF), and other fit indices, are commonly used to evaluate model fitness [89].

According to previous studies, it is suggested that the Comparative Fit Index (CFI) and Tucker-Lewis Index (TLI) should ideally exceed a threshold of 0.9. Simultaneously, the values of the Goodness of Fit Index (GFI) exceed the threshold of 0.8, indicating an acceptable level of fit [67]. The Root-mean-square error of approximation (RMSEA) is recommended to be less than 0.08 to indicate a satisfactory fit for the study model [90].

The parameters related to the current study model have been evaluated against these criteria and are presented in Table 6. By comparing the values of the fit indices to the established thresholds, the model’s goodness of fit can be assessed.

**Table 6:** Results of fit indices.

Index	Research model	Criteria
Chi-square (X^2^)	1284.57	——
DF	388	——
X^2^/DF	3.311	<5
CFI	0.895	>0.90
TLI	0.882	>0.90
GFI	0.818	>0.80
RMSEA	0.072	<0.08

### 4.5 Hypotheses tests

**Fig 2:**
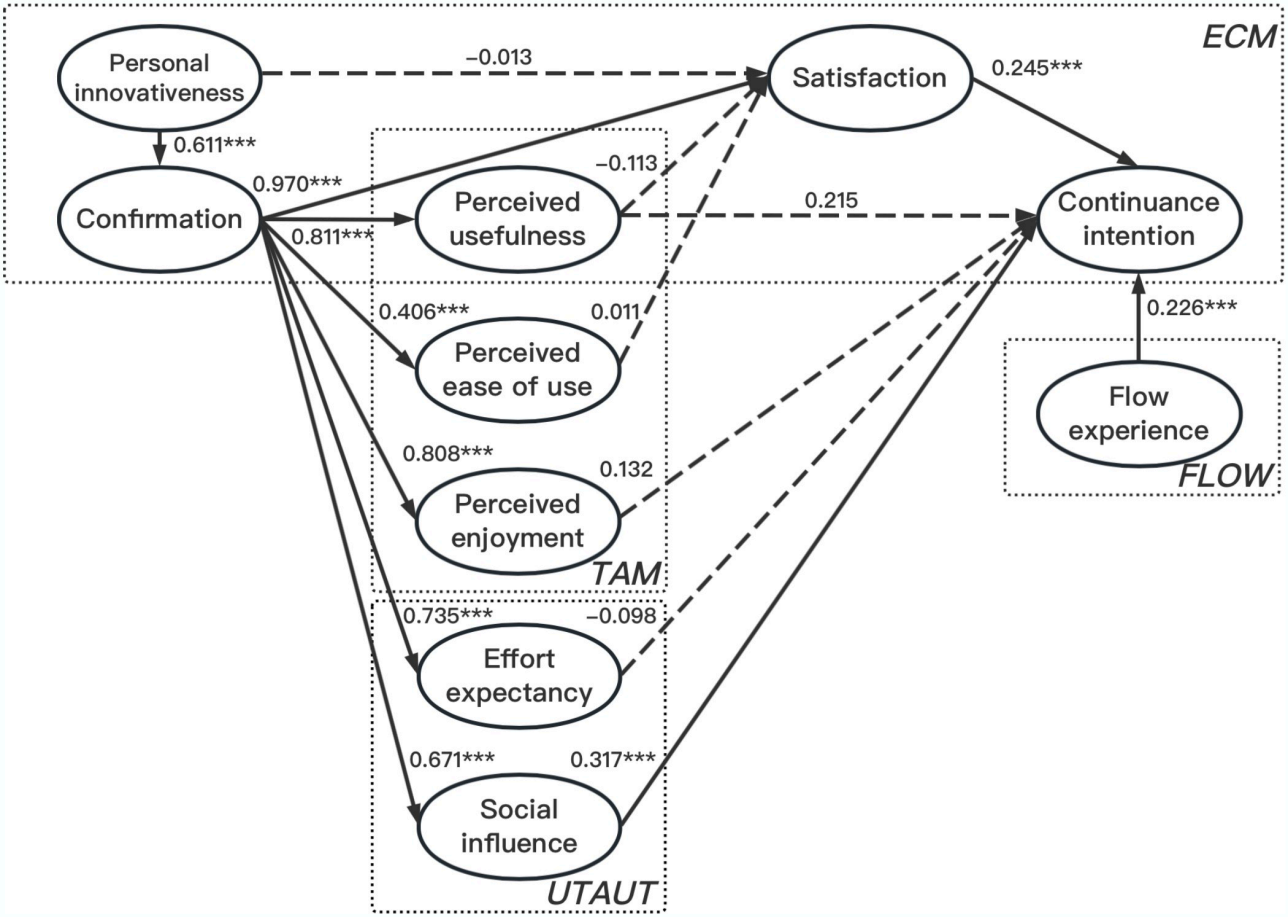
The results of the research model.

The results of the structural equation modeling (SEM) analysis conducted for the present study are summarized in Table 7, Figs 1 and 2.

**Table 7:** Results of the research mode.

Hypothesis	Relationship	Standardized Estimate(β)	Unstandardized Estimate	C.R (T-value)	S.E. (Standard Error)	P-Value	Decision
H1	SAT→ITU	0.245	0.265	3.616	0.073	***	Support
H2	CON→SAT	0.970	0.943	10.843	0.087	***	Support
H3	CON→PU	0.811	0.719	14.678	0.049	***	Support
H4	CON→PEOU	0.406	0.344	6.984	0.049	***	Support
H5	CON→PE	0.808	0.742	15.890	0.047	***	Support
H6	CON→PEX	0.735	0.765	15.237	0.050	***	Support
H7	CON→SI	0.671	0.400	8.890	0.045	***	Support
H8	PI→CON	0.611	0.731	11.111	0.066	***	Support
H9	PI→SAT	-0.013	-0.015	-0.286	0.052	0.775	Not Support
H10	PU→SAT	-0.113	-0.124	-1.521	0.081	0.128	Not Support
H11	PU→ITU	0.215	0.255	3.227	0.079	0.001	Not Support
H12	PEOU→SAT	0.011	0.013	0.295	0.044	0.768	Not Support
H13	PE→ITU	0.132	0.151	2.005	0.075	0.045	Not Support
H14	PEX→ITU	-0.098	-0.099	-1.751	0.057	0.080	Not Support
H15	SI→ITU	0.317	0.559	5.402	0.103	***	Support
H16	FLO→ITU	0.226	0.334	4.809	0.069	***	Support

Analysis of Fig 2 reveals that confirmation has a positive effect on various factors, including satisfaction to use (H2), perceived usefulness (H3), perceived ease of use (H4), perceived enjoyment (H5), effort expectancy (H6), and social influence (H7). These effects are supported by statistically significant t-values and beta coefficients, with specific values reported as follows (t = 10.843, β = 0.970, P<0.05 for H2; t = 14.678, β = 0.811, P<0.05 for H3; t = 6.984, β = 0.406, P<0.05 for H4; t = 15.890, β = 0.808, P<0.05 for H5; t = 15.237, β = 0.735, P<0.05 for H6; and t = 8.890, β = 0.671, P<0.05 for H7).

Additionally, personal innovativeness (H8) demonstrates a positive influence on confirmation, supported by a statistically significant t-value (t = 11.111, β = 0.611, P<0.05). However, personal innovativeness (H9), perceived usefulness (H10), and perceived ease of use (H12) do not show any significant influences on satisfaction with use.

Furthermore, satisfaction to use (H1), perceived usefulness (H11), perceived enjoyment (H13), social influence (H15), and flow experience (H16) positively impact continuance intention to use. These effects are supported by statistically significant t-values and beta coefficients (t = 3.616, β = 0.245, P<0.05 for H1; t = 3.227, β = 0.215, P<0.05 for H11; t = 2.005, β = 0.132, P<0.05 for H13; t = 5.402, β = 0.317, P<0.05 for H15; t = 4.809, β = 0.226, P<0.05 for H16).

However, it should be noted that effort expectancy (H14) does not exert any significant effect on continuance intention to use, as indicated by a non-significant t-value (t = -1.751, P>0.05).

### 4.6 The moderating effect analysis

In the present study, the PROCESS plug-in in SPSS was utilized to analyze H17, which examines the impact of habit on the correlation between social influence and continuance intention to use. This plugin, developed in 2013, facilitated the implementation of hierarchical regression analysis with bootstrapping, using a sample size exceeding 5000 iterations [83].

**Fig 3:**
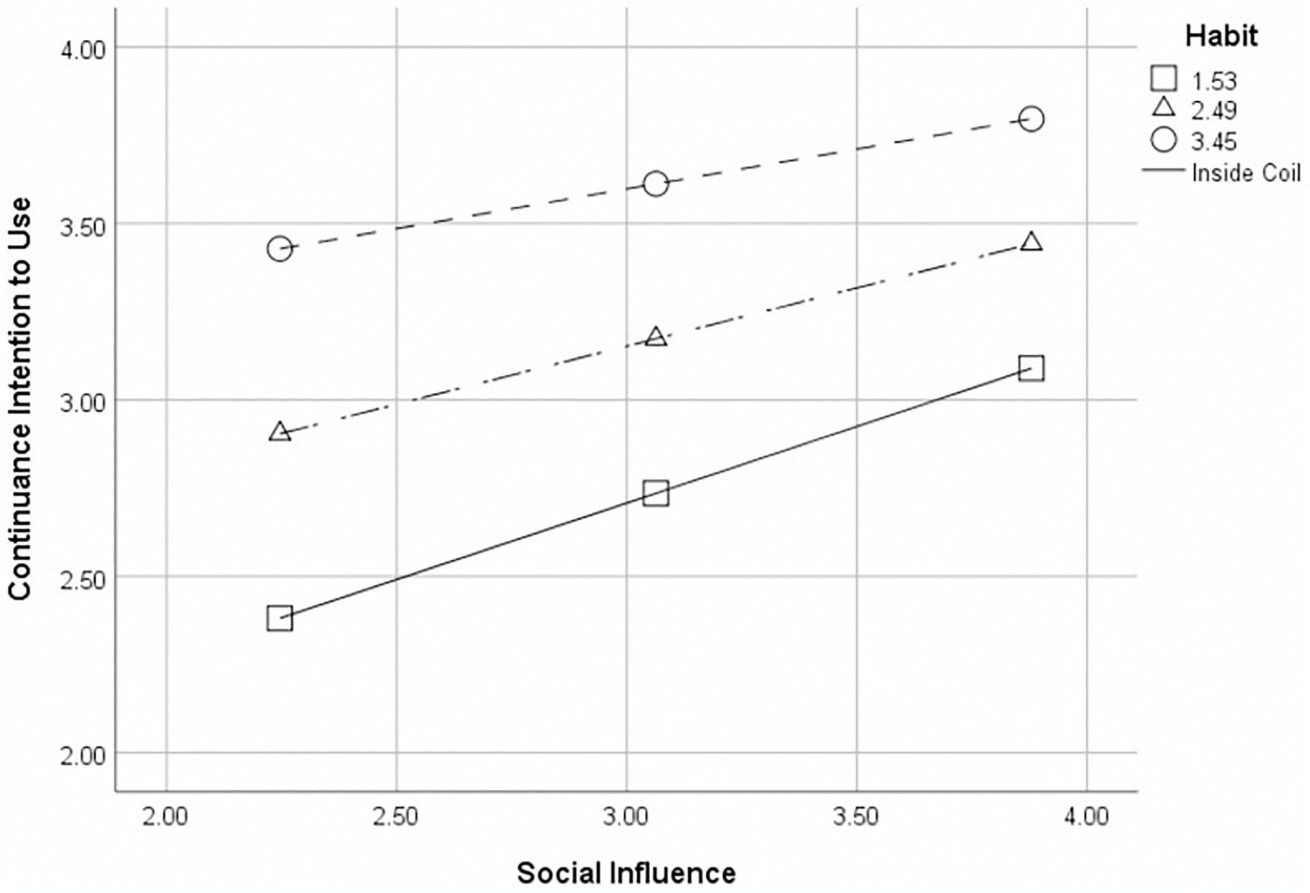
The moderation effect.

**Fig 4:**
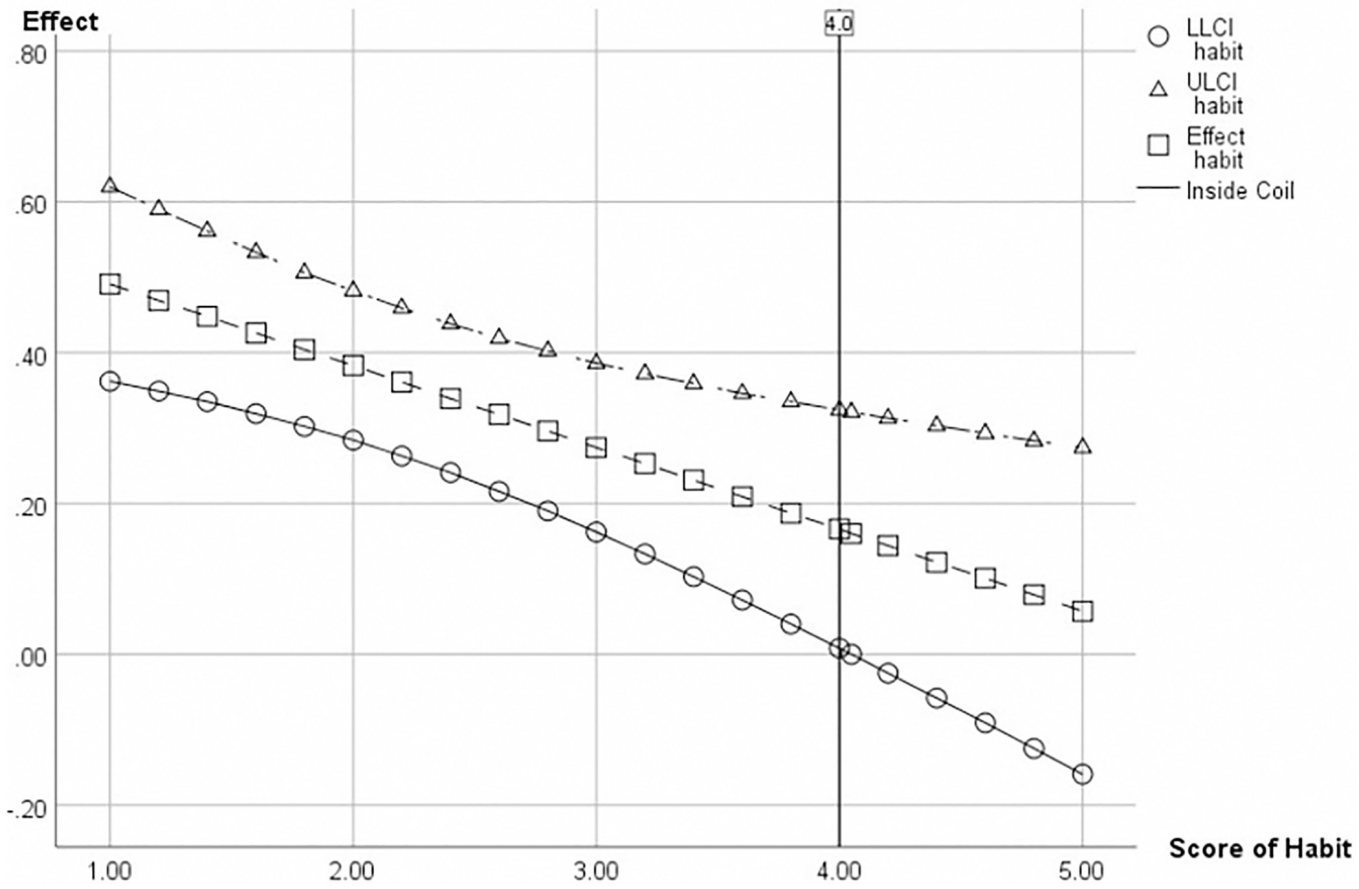
Johnson-Neyman confidence of moderating effect.

The P-value was used as an indicator to estimate the presence of a conditional effect. The results presented in Table 8 indicate that there is a moderation factor of habit in the conditional effect of social influence on continuance intention to use. The statistical analysis shows that the null hypothesis (p<0.05) can be rejected, suggesting a significant correlation between social influence and continuance intention to use. Additionally, the analysis reveals that this relationship is negatively moderated by the moderating variable, habit, as depicted in Fig 3. This implies that with the increasing effect of habit, social influence has a stronger influence on continuance intention to use. Conversely, when the effect of habit decreases, the association will be weaker. Thus, hypothesis H17 is supported. Furthermore, the study employed the Johnson-Neyman (J-N) technique to discern the superior effect from the inferior effect while conducting tests on basic slopes [91]. Fig 4 illustrates that there is no significant moderation between social influence and continuance intention to use for customers with habit scores above 4.0 (p>0.05). However, a notable conditional effect is observed when habit scores fall below a threshold of 4.0.

**Table 8:** Results of moderation effect.

Variable	Coeff	Se	T	P	LLCI	ULCI
HAB→ITU	0.798	0.13	6.159	0	0.543	1.053
SI→ITU	0.544	0.096	5.657	0	0.355	0.733
HAB X SI→ITU	-0.097	0.036	-2.701	0.007	-0.168	-0.027

## 5 Discussion and conclusions

This study aimed to develop a theoretical framework and investigate users’ intention to continue using an AI painting application by combining the Expectation Confirmation Model (ECM) with elements from TAM, UTAUT, and Flow Theory. The SEM analysis supported 11 out of 17 hypotheses, providing empirical evidence for most of the assumptions in the paper. The results of each hypothesis are discussed in detail below.

Firstly, the study unveiled that the majority of hypotheses concerning the influence of antecedents on satisfaction were supported. Users’ confirmation had a positive impact on perceived ease of use, perceived usefulness, perceived enjoyment, effort expectancy, social influence, and satisfaction. Confirmation plays a pivotal role, significantly and positively predicting satisfaction and social impact. Moreover, individuals’ innovativeness has a significant impact on confirmation. However, perceived usefulness, perceived ease of use, and personal innovativeness did not affect the intention of being satisfied with AI painting. This indicated that users’ expectations regarding utility, ease of use, and innovative potential might not have been met within the context of AI painting. The lack of impact on satisfaction could be attributed to the misalignment between users’ perceived expectations and their experience with AI painting tools. Additionally, for some individuals, the disparity between traditional artistic processes and the automated nature of AI painting tools could result in a feeling of dissatisfaction or disconnection [17]. Furthermore, the limitations and constraints imposed by AI technology in the creative process could also contribute to a lack of satisfaction among users who appreciate the depth and personal touch offered by traditional art forms [92].

In essence, the dissatisfaction could stem from a mismatch between users’ preconceptions about technology and their actual engagement with AI painting, as well as a desire for a more personalized and emotionally engaging experience [4]. The research findings indicate a lack of correlation between personal innovativeness and satisfaction with AI painting. Personal innovativeness typically involves individuals’ unique and forward-thinking creative abilities in a creative domain [40, 93]. When using AI painting tools, personal innovativeness may not directly impact satisfaction as these tools often incorporate algorithms and data to generate art pieces, and users primarily interact with the tool rather than create the entire piece. In this scenario, the scope for personal innovativeness to influence satisfaction is limited. Furthermore, the design and functionality of AI painting tools may constrain the expression of personal innovativeness. Some users may expect greater flexibility in incorporating their creativity and ideas, but the algorithms and operational methods of AI painting tools may limit the individual’s space for innovation, thereby weakening the correlation between personal innovativeness and satisfaction [61, 94].

Secondly, the findings indicated that users’ willingness to continue using AI painting was influenced by satisfaction, perceived usefulness, perceived enjoyment, effort expectancy, social influence, and flow experience. Particularly, satisfaction, flow experience, and social influence directly and positively predicted intention, with social influence demonstrating the most substantial impact. Surprisingly, it was found that effort expectancy had a relatively limited effect on continuance intention, in contrast to earlier research highlighting its significance in the success of app services [33, 34].

The research findings indicated that perceived usefulness, perceived enjoyment, and effort expectancy did not impact the intention to continue using AI painting. These results are not similar to those of Xu (2023) and Gupta (2021) [18, 31]. One possible explanation within the realm of artistic creation and innovative expression is that individual cognition and emotional experiences might play a more significant role. While perceived usefulness is typically a key factor influencing the acceptance and use of technology, in the context of art and creativity, individuals’ aesthetic experiences of the artwork itself could be more influential. The complexity of AI painting tools and the constraints they may impose on the creative process could also contribute to the lack of correlation with perceived enjoyment. Users may perceive AI-generated artwork as lacking the emotional depth or personal touch that traditional art forms provide, affecting their overall enjoyment of the AI painting activity. Additionally, effort expectancy may lose its impacting power on continuation intention in this context, as the pleasure and satisfaction derived from the artistic creation process for many individuals may outweigh the associated effort costs [95, 96]. Therefore, the study results indicate that in the realm of artistic creation, individuals value personal experiences and emotions more than practicality, enjoyment, or effort expectancy. This perspective could offer new insight into understanding people’s attitudes towards using AI painting technology, suggesting that in designing and promoting similar technologies, a greater emphasis on user experience and emotional needs, over and above the functional utility of the technology itself, may be required.

Finally, the study delved into the moderating role of habits in the relationship between social influence and the intention to continue using the AI painting application. The results indicated that habitual factors could interfere with the equilibrium between social influence and sustained usage. Put simply, as behavior becomes more ingrained as a habit, it is more inclined to have a negative impact on this association, consistent with previous research [8, 77].

### 5.1 Contributions

The main contribution of this study is the adaptation of the ECM framework for the context of AI painting, particularly within social media platforms. By expanding the applicability of ECM, we have enhanced our understanding of predictors of user satisfaction and intention to continue using AI painting. We have redefined the concept of usability to align with the unique features of AI painting technology and its intended goals on social platforms. Specifically, we introduced dimensions of effort expectancy, social influence, and perceived enjoyment to address these aspects. While previous studies have explored the role of effort expectancy and social influence on continuance intention to use [70, 74, 75], we extended this research to the context of AI painting. Our findings indicate that positive social influence can significantly influence a user’s intention to continue using AI painting.

Another significant aspect of expanding the ECM framework is the inclusion of personal innovativeness as a factor influencing confirmation and satisfaction. The original ECM assumes a passive user who either accepts or rejects the available technology, without considering alternatives. However, in the AI painting market, users have multiple options to choose from, and switching between applications incurs minimal costs [97]. Many users utilize multiple AI painting applications to engage with different groups of people. Additionally, recent developments have introduced features in AI painting applications that enable users to send and receive money, intensifying the competition among AI painting providers.

### 5.2 Implications

This study provides several implications for scholars and practitioners in the field of AI painting. Firstly, the study proposed AI painting continuance use model delineates a specific set of factors that service providers and application developers could make to enhance the continuous adoption and utilization of AI painting. It suggests that AI painting providers should explore interventions that increase the likelihood of positive confirmation and user satisfaction, as both factors significantly influence one’s intention to continue using AI painting. With the widespread use of AI painting tools, service providers should be more flexible and responsive to changes in the AI painting landscape, focusing on social influence, satisfaction, and flow experience within their AI painting applications.

Secondly, the results of the research model can be utilized by AI painting app service providers to enhance the user experience. They can apply the findings to improve perceived ease of use, perceived usefulness, perceived enjoyment, social influence, effort expectancy, and flow experience. Closing the gap between user expectations and actual usage experience by enhancing personal innovativeness is crucial for app service providers.

Thirdly, apart from continuously refining and improving the design of AI painting tools, technical developers should acknowledge that users may not be fully prepared for the capabilities offered by advanced AI tools, especially when lacking proper guidance. It is important to educate users about AI technologies through platforms like TikTok to increase their comfort and knowledge about AI tools and boost their overall AI painting experience.

Finally, the research findings indicate that users alter their decision-making patterns as they develop a habit of online AI painting.

### 5.3 Limitations and future work

While the current research has provided valuable insights into AI painting, it is important to acknowledge certain boundaries that should be addressed in future investigations. Firstly, the application of AI painting may vary among different age groups, genders, and backgrounds. However, the current study did not explore the potential differences resulting from age or gender. Therefore, future research could delve into this aspect and examine how disparities between different groups may lead to distinct outcomes.

Secondly, it is recommended for future studies to investigate the moderating effect of additional factors, such as experience and trust, on the relationship between user behavior and AI painting. These factors could potentially influence users’ perceptions and continuance intentions in using AI painting applications.

Lastly, the current research was confined to a single AI painting application within one nation, without considering other applications in the market. Therefore, further research is necessary to validate and generalize the findings by including multiple applications and comparing the results with the present study.

## Supporting information

S1 Dataset (XLS)
